# Contrast Agent-Free Assessment of Blood Flow and Wall Shear Stress in the Rabbit Aorta using Ultrasound Image Velocimetry

**DOI:** 10.1016/j.ultrasmedbio.2021.10.010

**Published:** 2022-03

**Authors:** Kai Riemer, Ethan M. Rowland, Jacob Broughton-Venner, Chee Hau Leow, Mengxing Tang, P.D. Weinberg

**Affiliations:** Department of Bioengineering, Imperial College London, London, United Kingdom

**Keywords:** Wall shear stress, Blood flow, Echo-particle image velocimetry, Ultrasound image velocimetry, Vector flow imaging, Blood speckle velocimetry, Native blood speckle, Atherosclerosis

## Abstract

Blood flow velocity and wall shear stress (WSS) influence and are influenced by vascular disease. Their measurement is consequently useful in the laboratory and clinic. Contrast-enhanced ultrasound image velocimetry (UIV) can estimate them accurately but the need to inject contrast agents limits utility. Singular value decomposition and high-frame-rate imaging may render contrast agents dispensable. Here we determined whether contrast agent-free UIV can measure flow and WSS. In simulation, accurate measurements were achieved with a signal-to-noise ratio of 13.5 dB or higher. Signal intensity in the rabbit aorta increased monotonically with mechanical index; it was lowest during stagnant flow and uneven across the vessel. *In vivo* measurements with contrast-free and contrast-enhanced UIV differed by 4.4% and 1.9% for velocity magnitude and angle and by 9.47% for WSS. Bland–Altman analysis of waveforms revealed good agreement between contrast-free and contrast-enhanced UIV. In five rabbits, the root-mean-square errors were as low as 0.022 m/s (0.81%) and 0.11 Pa (1.7%). This study indicates that with an optimised protocol, UIV can assess flow and WSS without contrast agents. Unlike contrast-enhanced UIV, contrast-free UIV could be routinely employed.

## Introduction

Cerebrovascular and coronary heart disease can be triggered by and cause local disturbances in blood flow and haemodynamic wall shear stress (WSS, *T*_w_) (Cecchi et al. 2011). For example, low, oscillatory and transverse WSS are all believed to be atherogenic and might predict sites of disease progression (Dhawan et al. 2010; Stone et al. 2012; Mohamied et al. 2014). High WSS likely plays a crucial role in aortic dilatation (Rodríguez-Palomares et al. 2018) because regions exposed to high WSS exhibit dysregulation of the extracellular matrix and medial elastin degradation. Intracranial aneurysms are more vulnerable to rupture when exposed to low WSS (Zhou et al. 2017). Thus, identifying regions of abnormal flow with high or low WSS could lead to a better understanding of the underlying pathology, identify high-risk areas and improve disease outcome.

WSS is the product of blood viscosity *µ* and the first-order spatial derivative of velocity (shear rate) near the wall. Its quantitative assessment *in vivo* is difficult. High spatiotemporal resolution, large dynamic range of detectable velocities and precise localization and tracking of the luminal boundary are required to accurately estimate the shear rate. The rheology can also be complex, but Newtonian rheology is often assumed for large arteries so that WSS reduces to

(1) $$T_{w}=\mu \frac{du}{dy}{|}_{y=0}$$

where *y* is the coordinate normal to the wall.

In principle, Doppler ultrasound systems can be used to determine flow velocity and hence WSS. WSS can be inferred from spectral Doppler by assuming Poiseuille or Womersley flow, and colour Doppler can determine flow and instantaneous velocity profiles (Markou and Ku 1991; Brands et al. 1995; Mynard et al. 2013). However, Doppler imaging is limited by angle dependency; velocity can only be measured along the beam direction. Finite aperture size and high-velocity gradients lead to spectral broadening, and errors increase with the beam-to-flow angle (Hoskins 2011). Angle-independent Doppler vector flow imaging (VFI) provides multidimensional velocity estimation, for example, through transverse oscillation, directional beam forming or synthetic aperture imaging (Jensen et al. 2016a, 2016b; Hansen et al. 2017). Numerous Doppler VFI techniques have been reported to measure the magnitude and direction of 2-D flow (Yiu et al. 2014; Collins et al. 2019), and a number of Doppler VFI methods have been implemented in commercial systems (Hansen et al. 2017). Technological advances have even led to volumetric Doppler VFI with 2-D arrays (Correia et al. 2016; Holbek et al. 2016; Wigen et al. 2018). Two-dimensional WSS has been measured in a carotid bifurcation albeit only at manually selected locations (Du et al. 2020). However, the imaging of deeper structures with vector Doppler techniques can suffer from reduced temporal or spatial resolution (Hansen et al. 2017). High flow velocities in the lateral direction or a low signal-to-noise ratio (SNR) can lead to aliasing inaccuracies (Goddi et al. 2017). Frame rates can also be too low for applications such as wave intensity analysis based on arterial diameter and blood velocity (Feng and Khir 2010; Rowland et al. 2020).

Plane wave contrast-enhanced ultrasound image velocimetry (CEUIV) correlates microbubble speckle patterns in consecutive B-mode images. High-frame-rate (HFR) CEUIV has been reported to accurately measure velocities near the moving arterial wall (Kim et al. 2004; Zhang et al. 2011; Poelma et al. 2012), and CEUIV can be used to estimate WSS accurately in 2-D (Gates et al. 2018; Leow and Tang 2018; Riemer et al. 2020a) and 3D (Riemer et al. 2020b). The use of microbubbles is particularly advantageous in regions where the blood signal is weak or the blood has a velocity similar to that of tissue. However, intravenous administration of contrast agents can be a significant limitation (Martin and Dayton 2014). Microbubble imaging can be restricted by maximum dosage in clinical applications, whilst in preclinical studies involving small animals, it can be difficult to find a suitable injection site, and even small quantities of injected fluid might change arterial pressure. Therefore, CEUIV is not routinely recommended (Jensen et al. 2016a).

Contrast agent-free UIV (CFUIV) comes at the expense of poor SNR (Trahey et al. 1987). Although the signal from red blood cells (RBCs) can be detected within the frequency range of medical ultrasound (Yu et al. 2009; Nam et al. 2012), it is comparatively weak. Nevertheless, recent advances in HFR imaging have permitted RBC speckle tracking in neonates (Fadnes et al. 2014), and contrast-free velocity estimation with blood-mimicking fluid has been reported (Voorneveld et al. 2016). The introduction of spatiotemporal filters - specifically singular value decomposition (SVD) - to HFR ultrasound significantly increases SNR (Demené et al. 2015) and can facilitate contrast-free RBC speckle tracking. Such advances could allow CFUIV to become a practicable technique, overcoming most of the disadvantages of both VFI and CEUIV.

Blood speckle intensity rises as the frequency or size of RBC aggregates increases. Aggregation is driven by low temperature, steady flow, low shear stress and a high plasma concentration of macromolecules (Lupotti et al. 2003). Three zones of echogenicity as a function of the shear rate have been reported, with larger aggregates and intensity in the center of the vessel (Cloutier et al. 1996). In pulsatile flow, long aggregates are less likely to occur (Nam et al. 2012), but temporal variation has been observed, with blood being most echogenic when flow is rapidly accelerating (Nguyen et al. 2008; Paeng et al. 2010). Whereas contrast agents spread evenly in the vessel. The influence of these characteristics on velocity and WSS estimation is unknown.

This study is the first to describe broad-view blood flow and WSS measurement with HFR CFUIV *in vivo*. We first simulate Womersley flow in a straight vessel to investigate the effects of radial intensity variation, SNR, scatterer density and aggregation on the accuracy of blood flow and WSS estimation. Subsequently, we optimize imaging parameters such as mechanical index (MI) and number of frames for achieving a high SNR after SVD-based clutter filtering. Next, we determine contrast agent-free blood flow and WSS measurement *in vivo* in the abdominal aorta of New Zealand White (NZW) rabbits and address radial and temporal changes in intensity. Finally, we assess the accuracy of blood velocity and WSS waveforms measured using CFUIV by comparing them with data obtained with an established CEUIV method *in vivo* in the aortas of five rabbits.

## Methods

### Flow simulation

To assess the effect of scatter properties on the accuracy of blood flow and WSS measurement, we modeled flow in the abdominal aorta of a NZW rabbit as Womersley flow with alterations in scatter amplitude, radial intensity distribution and scatter density. Simulated Womersley flow was based on a velocity waveform previously acquired in a rabbit abdominal aorta. The ultrasound acquisition of the flow was then simulated for a plane through the central axis using Field II (Jensen 1992, 1996).

### Womersley flow generation

Womersley found that by assuming a homogeneous, incompressible and Newtonian fluid in a rigid, cylindrical tube with no radial movement of the fluid, the Navier–Stokes equations can be simplified by neglecting the non-linear terms. Following He et al. (1993), a periodic cross-sectional mean velocity waveform $\left({\hat{V}}_{\text{uiv}}\right)$ can be expressed as the sum of a Fourier series with *n* harmonics.

(2) $${\hat{V}}_{\text{uiv}}=\text{Real}\left\{\sum_{j=0}^{n}{\hat{V}}_{j}e^{ijwt}\right\}$$

The corresponding velocity profile can be found by the inverse Womersley method as

(3) $$u\left(r,t\right)=\text{Real}\left\{\sum_{j=0}^{n}{\hat{V}}_{j}\left\{\frac{1-J_{0}\left(i^{3/2}\alpha_{j}r/R\right)}{J_{0}\left(i^{3/2}\alpha_{j}\right)\left(1-\frac{2J_{1}\left(i^{3/2}\alpha_{j}\right)}{i^{3/2}\alpha_{j}\left(i^{3/2}\alpha_{j}\right)}\right)}\right\}e^{ijwt}\right\}$$

where ${\hat{V}}_{j}$ is the complex coefficient of the *j*th harmonic of the mean waveform, J_m_ the *m*th-order Bessel function of the first kind, *R* the vessel radius, *r* the radial position from 0 to *R* and *t* the time. The Womersley number of the *j*th harmonic is denoted by $\alpha =R\sqrt{\omega_{j}/\nu }$, where $\omega_{j}$ is the angular frequency of the *j*th harmonic, and *v* is the kinematic viscosity. To accurately capture the original input waveform, *n* = 8 was sufficient. To introduce more retrograde flow, 0.1 m/s was uniformly subtracted from the measured cross-sectional waveform, prior to decomposition. To accommodate the difference between a parabolic and paraboloid mean, the velocity of the measured waveform was scaled by a factor of 0.7. The Fourier coefficients of the flow waveform and their corresponding frequencies and Womersley numbers are outlined in Table 1.

**Table 1:** Fourier components of the waveform in the abdominal aorta of a New Zealand White rabbit

n	f	α	${\hat{V}}_{j}$
0	0	-	1
1	3.32	4.64	1.54
2	6.64	6.56	0.94
3	9.96	8.04	0.72
4	13.29	9.28	0.29
5	16.61	10.38	0.17
6	19.93	11.37	0.10
7	23.25	12.29	0.06
8	26.58	13.13	0.03

### Ultrasound simulation

A Verasonics 128-element L11-4v equivalent ultrasound imaging scheme was simulated. The temporal resolution was 2.2 × 10^–4^ s per time step, equivalent to a pulse repetition frequency of 4500 Hz. The total duration of the simulation was a single cardiac cycle corresponding to 0.3 s or 450 high-resolution frames composed of three plane waves, having an angle range of 12° (–6°, 0°, 6°). The vessel was centred at a depth of 14 mm with a diameter of 4 mm. The wall was modelled as a single 200-*µ*m-thick layer containing 20 scatterers per resolution cell, with constant amplitude. Flow was simulated via randomly distributed point scatterers with a normally distributed base amplitude centred at 0. The position of each scatterer was updated at each time step. Random Gaussian noise was added to each simulation, yielding a SNR of 12 dB based on the wall signal as reference. The base amplitude of scatterers was scaled between 0.1 to 10, giving an SNR of 1.6, 13.5, 22.3 or 44.7 dB in the flow signal after clutter filtering. Flow scatterer density per resolution cell was altered from 0.1 to 1000. The radial intensity was kept constant or decreased linearly or as a power of 2 or 4 with increasing distance from the centre line (Paeng et al. 2010). When any of these scatter variables were altered, the other variables were set to default values. A complete list of simulation parameters is provided in Table 2 (default values in bold).

**Table 2:** Field II simulation setup and scatter properties*

Centre frequency	6.25 MHz	Transmit frequency	8 MHz
No. of elements	128	Element pitch	3.00*e*-4 m
Element width	2.7*e*-4 mm	Element height	5 mm
Sampling frequency	25 MHz	Elevational focus	18 mm
Number of subapertures	4	PRF	4500 Hz
Number of angles	3	Angles	–6, 0, 6
Scatter properties			
Mean wall amplitude factor		20	
Wall scatter density (per resolution cell)		20	
Normal distributed flow amplitude factor		0.1, 0.5, 1, **10**	
Flow scatter density (per resolution cell)		0.1, 1, **10**, 100	
Radial variation factor		**Constant**, r, *r*^2^, *r*^4^	

PRF = pulse repetition frequency.

⁎ Boldface defines the default value used when varying other scatter variables.

### In vivo imaging of the rabbit abdominal aorta

#### Experimental protocol

HFR plane-wave images of the abdominal aorta of five male New Zealand White rabbits (HSDIF strain, specific pathogen-free, mean age: 12 weeks; mean weight: 2.69 kg; Envigo UK) were obtained using a Verasonics Vantage 128 LE research ultrasound system (Kirkland, WA, USA) and a linear L11-4v broadband probe. All experiments complied with the Animals (Scientific Procedures) Act 1986 and were approved by the Animal Welfare and Ethical Review Body of Imperial College London. Animals were housed individually in pens on a 12-h day–night cycle and fed a standard laboratory diet. Water was given *ad libitum*. Following sedation with acepromazine (0.5 mg/kg, i.m.), rabbits were anaesthetised with medetomidine (0.25 mL/kg, i.m.) plus ketamine (0.15 mL/kg, i.m.) and maintained with one-third of these doses every 30 min. The rabbits were ventilated after tracheotomy at 40 breaths/min. A rectal probe and heating mat were used to monitor and control body temperature. For imaging, the animals were turned on their backs, and fur from below the region of the rib cage was shaved. To ensure comparability of measurements, the position of the ultrasound probe was fixed with a clamp. Measurements were triggered by the mechanical ventilator.

#### Contrast agents

Decafluorobutane microbubbles (MBs) were prepared at a concentration of 5 × 10^9^ MBs/mL and 1-*µ*m average diameter (Sennoga et al. 2012). Dipalmitoylphosphatidylcholine (DPPC), 1,2-Bis(diphenylphosphino)ethane–polyethylene glucose 2000 (DPPE–PEG-2000) and chloride salt (16:0 1,2-dipalmitoyl-3-trimethylammonium-propane [TAP]) were dissolved in a molar ratio of 65:5:30 and total lipid concentrations of 0.75, 1.5 and 3 mg/mL in an excipient liquid composed of 15% propylene glycol, 5% glycerol and 80% normal saline (Sheeran et al. 2011). A 2-mL vial was filled with 1.5 mL of this solution, and microbubbles were formed by 60 s of agitation.

#### Plane wave imaging

Images were acquired at a pulse repetition frequency of 4500 Hz with three angles spanning 12° (–6°, 0°, 6°). The central transmit frequency was 8 MHz with an MI between 0.05 and 0.33. MI values were calibrated in a water tank. Except where the effect of MI was being studied, a low MI was used for contrast-enhanced imaging to avoid microbubble destruction (MI = 0.14, based on previous studies). For contrast-free imaging, the maximum MI was used (MI = 0.33, limited by transmission voltage). Contrast-free imaging was performed before contrast-enhanced imaging. Microbubbles were injected via the marginal ear vein in boluses of <25 µL/kg up to a total of 0.6 mL/animal. No contrast-specific acquisition scheme was used. The radiofrequency data were beamformed using an in-house delay-and-sum beam former. Where data sets were being compared, lower frame rates and numbers of frames were reconstructed from a subsample of the original acquisition. Further analysis was performed in MATLAB (The MathWorks, Natick, MA, USA).

#### Singular value decomposition

SVD clutter filtering was applied to each low-resolution image stack. Cutoff points were selected manually based on the spatial similarity matrix (Baranger et al. 2018) using the MATLAB function corrcoef(U).

#### UIV algorithm and WSS measurement

A purpose-written 2-D echo-particle image velocimetry (echo-PIV) algorithm was used to track the local displacement of scatterers in two consecutive images; coupled with the known time difference between the images, a velocity field could be calculated. The algorithm employed an iterative window deformation cross-correlation method (Leow and Tang 2018; Riemer et al. 2020a). In three iterations, the interrogation window size was halved, from 32 to 8 pixels with an overlap of 50%. Window deformation and a Gaussian subpixel estimator were used to improve accuracy of the displacement estimation. Outliers were filtered based on median spurious outlier detection, and the velocity field was ensemble averaged over 11 consecutive frames. A final regularization of the high-resolution velocity field was performed using proper orthogonal decomposition (POD). Table 3 lists all parameters used for the analysis.

**Table 3:** Settings of UIV algorithm

No. of iterations	3	Window size	32 pixels
No. of frames ensemble averaged	11	Window overlap	50%
Window deformation	Spline	No. of POD modes	10
Universal outlier detection	1.5	Relative SG filter length	0.4
Subpixel estimator	Gaussian	Lumen mask	LSS + DPF
Lateral resolution	75 µm	Axial resolution	52 µm

POD = proper orthogonal decomposition; SG = Savitzky–Golay; LSS = Level Set Segmentation; DPF = directional peak fitting.

The wall shear rate (WSR) was derived from the velocity profile along the wall normal. Specifically, the WSR was calculated from the two points closest to the wall of a third-order Savitzky–Golay-filtered velocity profile. The position of the wall was determined by the sparse field method (SFM, MATLAB file exchange: sfm-chanvese) (Lankton 2009) and subsequent directional peak fitting (DPF) (Riemer et al. 2020a) to accommodate interfaces in ultrasound imaging produce echoes that are much larger than their physical size (Wikstrand (2007). First, a contour image was created by subtracting the normalised flow signal from the normalised clutter signal obtained by SVD; both had been spatially smoothed with a Gaussian smoothing kernel (σ = 1.5) and a moving-window average. In the contour image, the SFM was used to trace the lumen boundary by solving for the signed distance function near the zero-level set (active contour segmentation based on Chan–Vese energy). In a subsequent step, the trace from SFM was expanded by peak fitting to the closest intensity peak of clutter signal within a 20-pixel distance. The expansion was performed pixel by pixel, based on a signed distance map (MATLAB: bwdist) in the outward direction. The integrity of the shape of the contour was maintained by a rolling average of the displacement of neighbouring pixels. An empirically determined displacement correction factor (k = 1_top_, k_bottom_ = 0.6) was used to account for the difference in the leading edge of the top and bottom wall (Wikstrand 2007). The estimated WSR was median filtered by its neighbouring values (MATLAB: medfiltl). Measurements were taken over at least three cardiac cycles, the results being aligned by the negative peak of each waveform.

#### Quantitative evaluation

In simulations, the errors in velocity, WSS and flow angle were calculated and averaged over the full acquisition; velocity and WSS errors were normalised by the peak velocity and peak WSS, respectively. SNR was averaged over multiple cardiac cycles. The ratio of clutter to signal intensity is defined as

(4) $$\text{SNR}=20\cdot \log\left(\frac{S_{\text{flow}}}{S_{\text{tissue}}}\right)$$

*In vivo* CEUIV and CFUIV results were assessed spatially and temporally. For the point-by-point comparison, bias of velocity magnitude and angle and the difference and correlation coefficient of WSS magnitude were calculated. To compare the level of agreement between CEUIV and CFUIV waveforms, a Bland–Altman plot, the root mean square error (RMSE) and the peak normalised relative difference were calculated.

## Results

### Womersley flow simulation

**Fig. 1:**
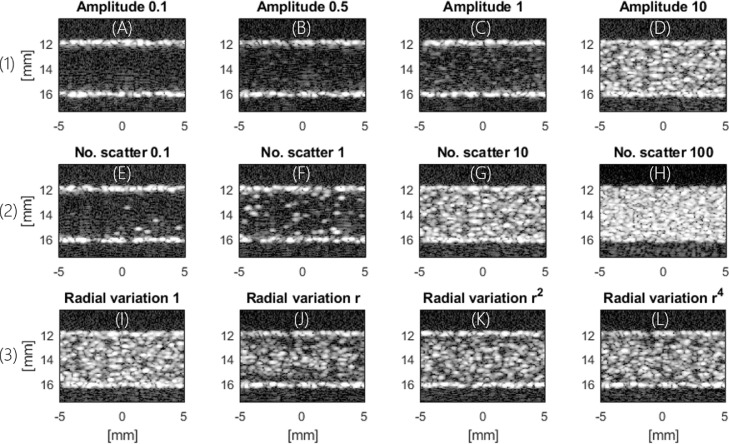
Womersley flow simulated with different parameters. (A–D) Variation in relative scatter amplitude to investigate the impact of signal-to-noise ratio (SNR). (E–H) Number of scatterers per resolution cell to investigate the impact of blood speckle intensity and aggregation. (I–L) Radial variation to investigate the impact of non-uniform intensity across the vessel. D, G and I are the same simulation.

**Fig. 2:**
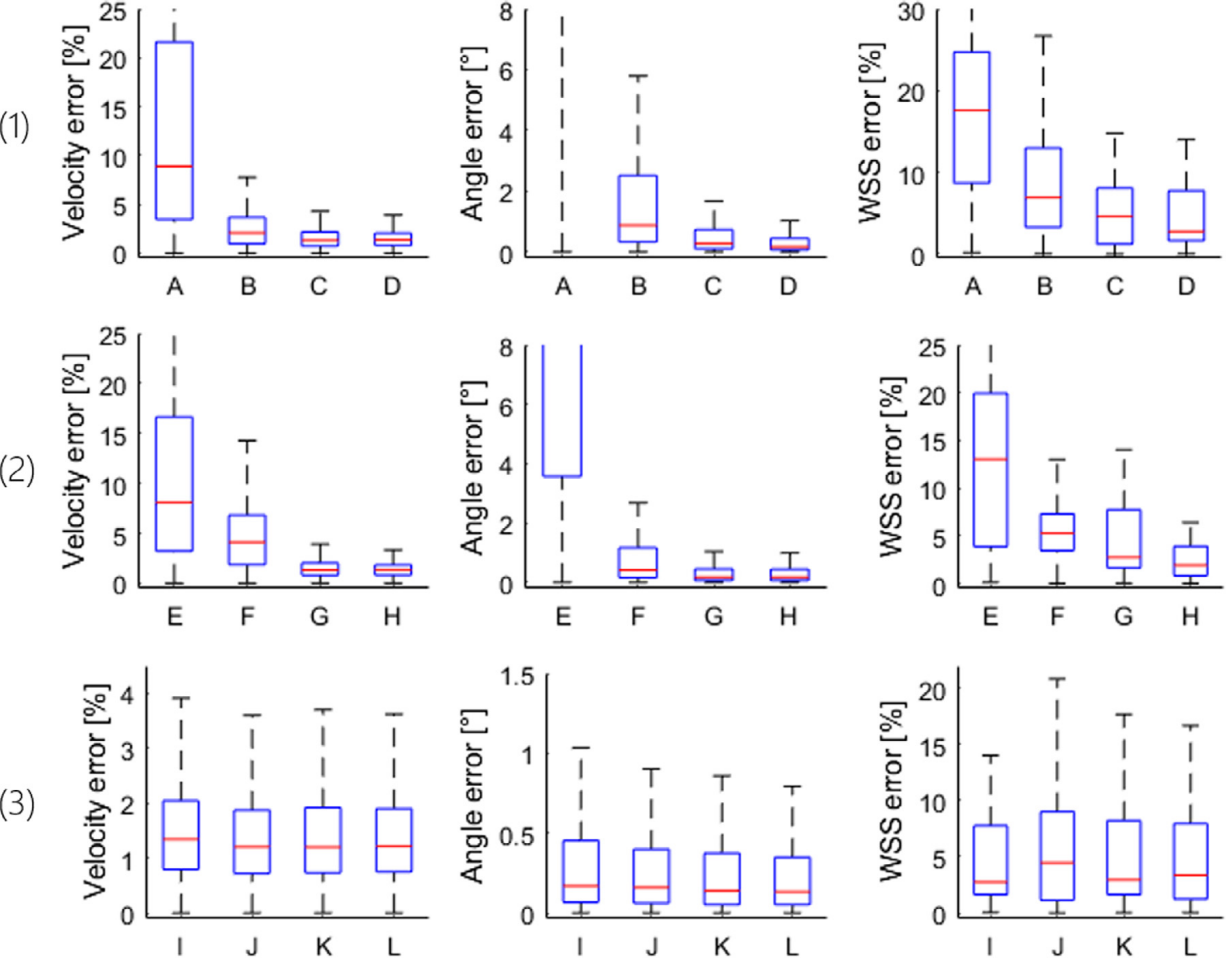
Percentage error in velocity and wall shear stress (WSS) magnitude and error in their angle. The letters under the boxplots correspond to the letters under the example images in Figure 1, which also illustrates which parameters were varied: (1) Scatter amplitude (low-to-high signal-to-noise ratio). (2) Scatter density (low-to-high density). (3) Radial intensity (uniform to uneven). Note that the y-axis limits are altered between plots.

The effect of scatter attributes on the appearance of B-mode images is illustrated in Figure 1. Figure 2 illustrates the corresponding percentage error in velocity, WSS magnitude and their angle.

The change in relative scatter amplitude was used to investigate the effect of SNR. Higher signal amplitude gave more accurate estimates of velocity magnitude, WSS magnitude and angle, as illustrated in Figure 2, row 1. A low median error of 2.5% in velocity and 7% in WSS magnitude were obtained for SNR values as low as 13.5 dB. The number of scatterers per resolution cell was used as a proxy for the degree of RBC aggregation and density. Its effect on velocity and WSS magnitude and angle is shown in Figure 2, row 2. The median WSS error was smallest (1.9 %) for 100 scatterers per resolution cell. Radial variation in intensity was used to mimic the dependence of aggregation and hence of scattering on shear rate. This may represent the biggest difference between using contrast agent or RBC speckle. WSS estimation was most accurate for a uniform intensity distribution, with 2.7% error, and least accurate for a linear radial decrease in intensity, with 4.4% error, as illustrated in Figure 2, row 3.

### Optimization of imaging parameters in the rabbit abdominal aorta

**Fig. 3:**
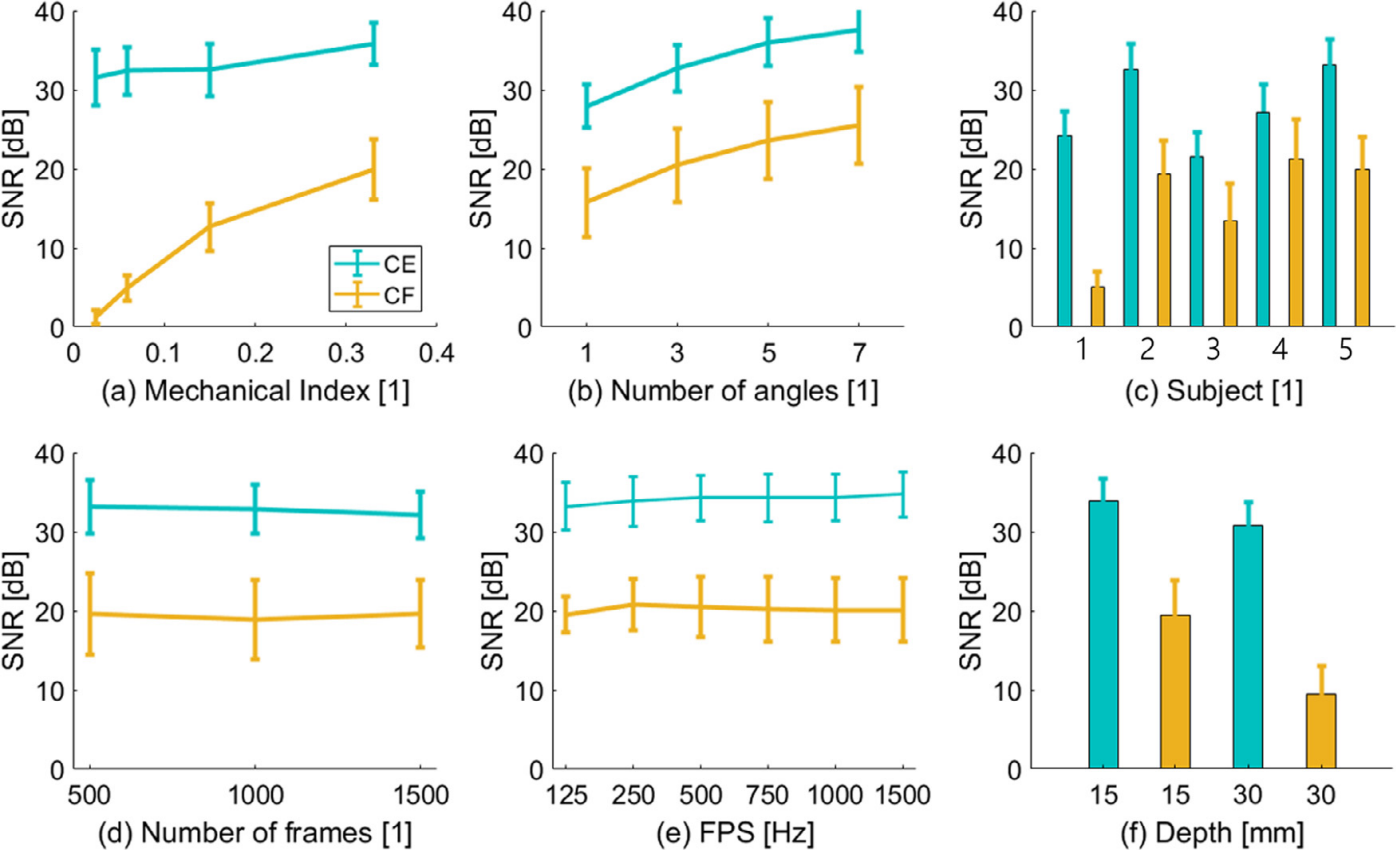
Impact of imaging parameters on signal-to-noise ratio (SNR) in images of the rabbit abdominal aorta. (a) Influence of mechanical index (MI) on SNR repeated for three cardiac cycles. (b) Influence of compounding angles on SNR. (c) Comparison of SNR between different animals (labeled 1–5) at same imaging depth and at a mechanical index (MI) of 0.14 for contrast-enhanced ultrasound image velocimetry (CEUIV) and 0.33 for contrast-free UIV (CFUIV). (d) Influence of singular value decomposition (SVD) stack size on the SNR at MIs of 0.14 for CEUIV and 0.33 for CFUIV in the same rabbit. (e) Influence of frame rate on SNR at MIs of 0.14 for CEUIV and 0.33 for CFUIV in the same rabbit. (f) Dependence of imaging depth on SNR at MIs of 0.14 for CEUIV and 0.33 for CFUIV in the same rabbit.

To minimize error in velocity and WSS magnitude, SNR must be maximised. Figure 3 illustrates the impact of MI, the number of compounding angles, the number of frames, frame rate and imaging depth on SNR after SVD clutter filtering. Variation between animals is also illustrated. SNR was averaged over 1 s, which corresponds to approximately three cardiac cycles. Figure 3a illustrates the SNR at a reference depth of 15 mm and a transmit frequency of 8 MHz. Figure 3b illustrates that SNR increases with the number of compounding angles. Figure 3c illustrates that SNR varied significantly between animals and in some cases was below the 13.5-dB threshold that simulations revealed were required for accurate velocity and WSS estimation. The minimum frame rate tested was 125 Hz, and at least one complete cardiac cycle was captured. The number of frames (Fig. 3d) and frame rate (Fig. 3e) had little impact on SNR. Figure 3f indicates that increasing imaging depth reduced SNR in the same animal.

### Blood flow and WSS assessed by CFUIV in the rabbit abdominal aorta

#### Maps of velocity and WSS

**Fig. 4:**
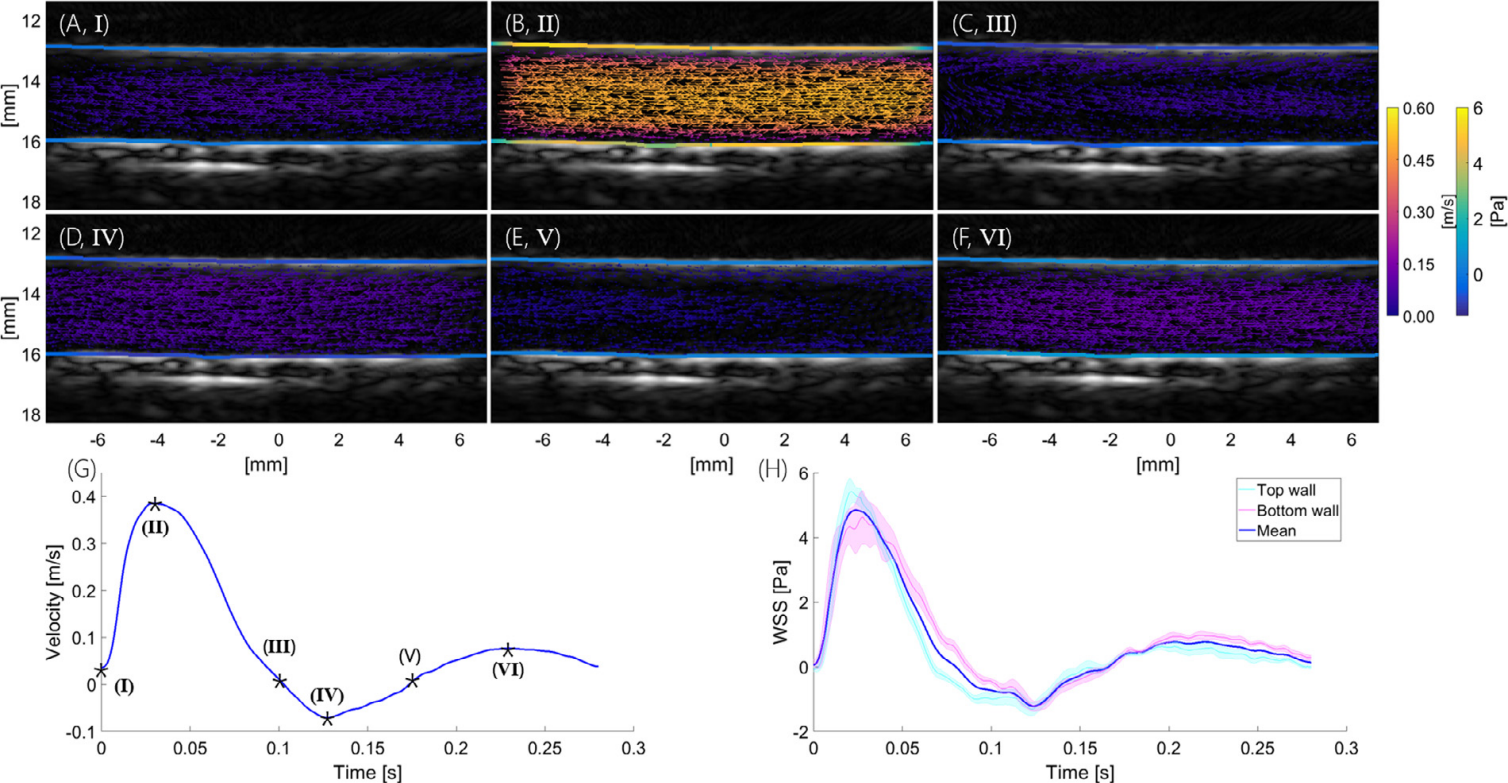
Example of in vivo contrast-enhanced ultrasound image velocimetry (CFUIV). (a–f) Velocity vector field and wall shear stress (WSS) in the abdominal aorta for different points during the cardiac cycle (I–VI). Graphs below illustrate mean velocity (g) and WSS (h) waveforms. Time stamps I–VI correspond to images (a)–(f). *Pink and teal lines* represent mean WSS at top (13 mm) and bottom (16 mm) wall with standard error (*shading*). The *blue line* represents overall mean WSS.

Figure 4a–f illustrates the velocity vector field and WSS in the abdominal aorta of a NZW rabbit at different points during the cardiac cycle. Mean velocity and WSS waveforms are also shown. WSS values on the top and bottom luminal boundary were very similar throughout the cardiac cycle. Peak WSS during systole was around 5 Pa, with peak velocities up to 0.6 m/s.

#### Radial and temporal changes in intensity

**Fig. 5:**
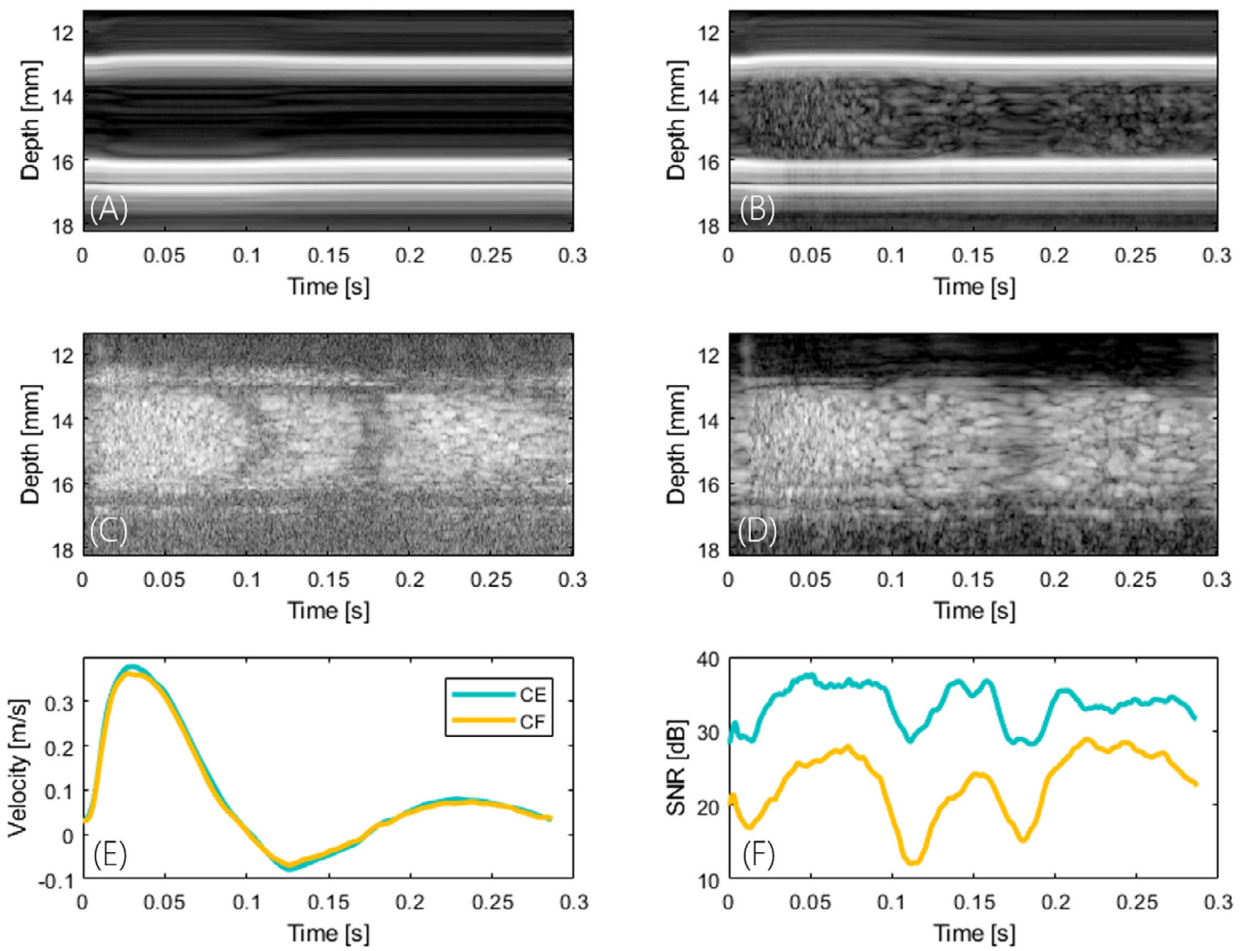
Radial and temporal changes in intensity. (a, b) B-Mode and (c, d) singular value decomposition-filtered intensity of the centre line over time. The contrast agent-free acquisition is displayed on the left (a, c) and the contrast-enhanced acquisition is shown for comparison on the right (b, d). (e) Cardiac cycle (corresponds to Fig. 4) and (f) temporal changes in signal-to-noise ratio during the cardiac cycle. Intensity varies spatiotemporally with the minimum occurring at around zero net blood flow velocity.

Radial and temporal changes in intensity occurred during the cardiac cycle. Figure 5 illustrates the central scan line over time for the unfiltered (a, b) and SVD-filtered (c, d) data sets, respectively. Changes in intensity as a function of radial distance and phase of the cardiac cycle for the contrast agent-free acquisition are displayed on the left (Figure 5a, 5c), and the contrast-enhanced acquisition is provided for comparison on the right (Figure 5b, 5d). Figure 5e illustrates the corresponding velocity waveform, and Figure 5f, the temporal changes in SNR during the cardiac cycle; the minimum occurs at around zero net blood flow velocity.

### Comparison of CFUIV and CEUIV assessments of blood flow and WSS in the rabbit abdominal aorta

#### Point-by-point comparison

**Fig. 6:**
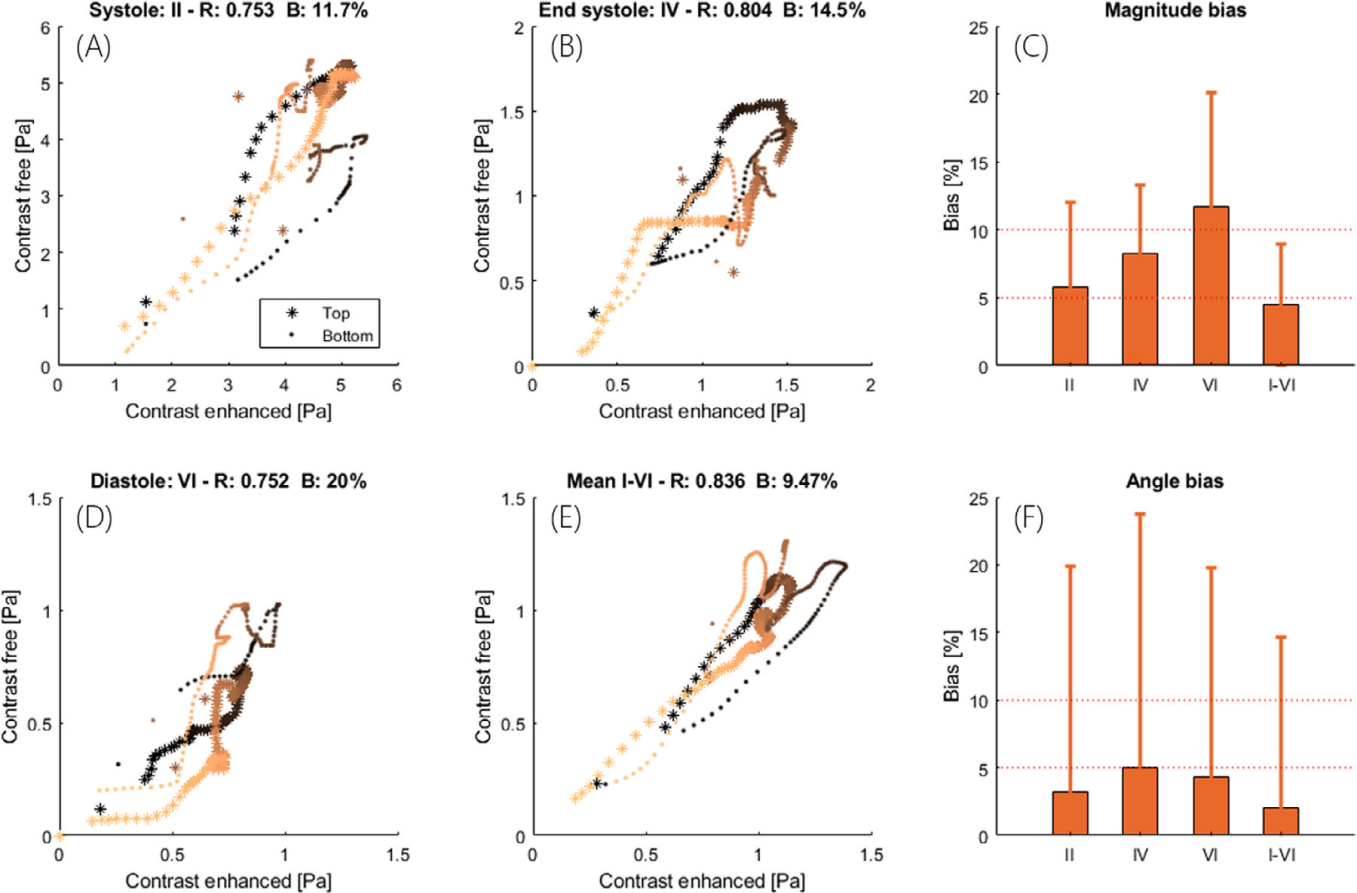
Comparison of contrast-enhanced ultrasound image velocimetry (CEUIV) and contrast-free UIV (CFUIV) determination of instantaneous and mean velocity and wall shear stress (WSS). (a–d) Scatterplot, correlation coefficient and WSS bias. (e, f) Bias in velocity magnitude and angle for three different points during the cardiac cycle and the cycle average. Scatterplot is color-coded to distinguish between top (*) and bottom (•) wall and left (bright) and right (dark) sides of the image. Data correspond to the rabbit in Figures 4 and 5.

The scatterplots in Figure 6a, 6b, 6d and 6e compare WSS obtained by CEUIV and CFUIV at three different points in the cardiac cycle and the cycle average. Figure 6c and 6f illustrate the bias in velocity magnitude and angle. Systolic, end-systolic and diastolic time points correspond to Figure 4 (roman letters). The mean point-by-point difference was 4.4% for velocity and 9.5% for WSS. The instantaneous difference during systole was 5.7% for velocity and 11.7% for WSS. Pairwise correlation coefficients between WSS measurements were between 0.75 and 0.83. High velocity angle bias coincided with the largest difference in WSS measurements.

#### Mean waveform comparison

**Fig. 7:**
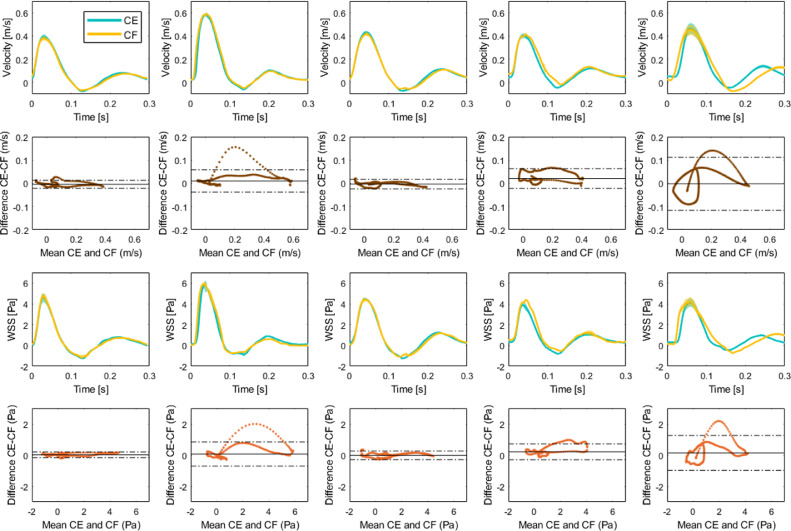
Variability of velocity and wall shear stress (WSS) waveforms measured *in vivo* in five New Zealand White rabbits, averaged over the image region and three cardiac cycles. Each column corresponds to a single animal. In the first and third rows are velocity and WSS waveforms acquired with contrast-enhanced ultrasound image velocimetry (CEUIV) or contrast-free UIV (CFUIV). In the second and fourth rows are corresponding Bland–Altman plots for velocity and WSS. *Dashed lines* mark ±1.96 standard deviation, indicating the level of agreement between CEUIV and CFUIV.

Each column of Figure 7 corresponds to a single animal. The first and third rows indicate velocity and WSS waveforms acquired with CEUIV or CFUIV. In the second row are the corresponding Bland–Altman plots for velocity and WSS. Waveforms were averaged over three cardiac cycles and peak aligned. The average SNR after clutter filtering was 31.29 ± 6.01 dB in CEUIV images and 19.41 ± 6.52 dB in CFUIV images. A high correlation between measurements of flow and of WSS waveforms were observed. For velocity, the largest RMSE was 0.03 m/s (3.8%), and the lowest RMSE was 0.01 m/s (0.81%). For WSS, the difference was generally higher - between 0.11 and 0.33 Pa (1.7% and 4.8%). The two methods agreed during systole and diastole but the level of agreement decreased towards the point of zero velocity.

## Discussion

A tool for accurately assessing haemodynamic WSS *in vivo* would be of value in the clinic and for investigating cardiovascular mechanics in general, but developing such tools remains challenging. CEUIV has been reported to measure spatially and temporally varying WSS in vivo with high accuracy (Riemer et al. 2020a) and has been used in clinical settings (Gates et al. 2018). The use of contrast agents, however, is limited by maximum permissible dosage, patient discomfort and limited examination times, and may lead to unwanted biological effects. In preclinical studies involving small animals, intravenous injection can require sedation or immobilization and may alter circulating fluid volume. In this study, we determined that CFUIV is capable of measuring spatially and temporally varying velocity and, hence, of assessing WSS, in large straight vessels *in vivo*. CFUIV waveforms agreed well with CEUIV, giving a correlation coefficient of up to 0.99 both for velocity and WSS. CFUIV might be used more broadly than CEUIV and with similar accuracy if a sufficient SNR can be achieved.

This development permits novel applications, such as pulse wave intensity analysis, which requires simultaneous measurement of flow velocity and wall displacement at frame rates higher than can be achieved with Doppler VFI (Goddi et al. 2017), or the assessment of arterial pathology and risk from locally elevated WSS. Commercial Doppler VFI can now estimate WSS locally and in real time (Collins et al. 2019; Du et al. 2020) but measurements are limited by the aperture size. CFUIV may prove more accurate and reliable for cardiac imaging or in situations where there is a high dynamic range of velocities and/or substantial lateral flow.

### Error attributed to scatter properties in simulation

Simulations revealed that the relative amplitude of scatterers, and hence the SNR, has the biggest impact on the accuracy of flow and WSS estimation (Fig. 2). The error was smaller for velocity magnitude than for velocity direction or WSS. Unless scatterers were very sparse, velocity and WSS estimation were insensitive to scatter density. Radial variation of intensity had only a moderate impact on median WSS but a more significant impact on the spread of locally determined WSS values, with a linear radial decrease in intensity within the vessel giving the worst results. Although *r*^2^- and *r*^4^-dependent radial decreases in intensity might appear more extreme, they create blunt distributions that are more like the uniform case. The small change of error with radial distribution and scatter density suggests that the effect of RBC aggregation on WSS estimation is minor.

### Imaging parameters in the rabbit abdominal aorta

The imaging parameter that most affected SNR in a large vessel with pulsatile flow was the MI (Fig. 3). It was limited to 0.33 in the present study solely for practical reasons (maximum of 40 V transmission) and should be as high as safety considerations allow. Frame rate and the number of frames did not significantly alter the SNR within the ranges tested, which is plausible for this type of flow. CFUIV acquisitions are susceptible to attenuation and have higher variability than CEUIV. This probably explains the depth-related reduction in SNR. CFUIV benefits from a large number of compounding angles. However, angle incoherence in areas of fast blood flow will limit the number of compounding angles that can be used.

### Measurement of velocity and WSS in the rabbit abdominal aorta

We previously reported a bias in estimated WSS between the top and bottom walls of the vessel (Riemer et al. 2020a). Methodological improvements in measuring the lumen diameter and the differences in location of the leading edge (correction factor) were implemented (Wikstrand 2007), and eliminated this error. Cyclic and radial variation in B-mode images were observed in the SVD clutter filtered images (Figure 5), as described before (Cloutier et al. 1996; Nguyen et al. 2008). The radial variation in intensity appears to be a function of scatterer velocity. Particles near the wall slow down faster than those in the centre as the flow decelerates. The SVD filter removes some of the slow-moving scatterers near the vessel boundary (Brown et al. 2019), which manifests itself in the shape of a protruding cone over time. The decrease in SNR was much higher for the contrast-free acquisition than with the microbubble acquisition, which suggests that shear-dependent aggregation and a decrease in RBC density towards the cell-free layer may make an even bigger contribution to the loss in intensity. Effects of temporal variations might be reduced with a sliding window SVD filter (Badeau et al. 2004). From the simulation results, the error in velocity and WSS measurement is expected to increase during diastole. This is seen in the differences between waveforms of velocity and WSS assessed by CFUIV and CEUIV during diastole, with CEUIV more likely to be more accurate.

CFUIV and CEUIV measurements cannot be made in the same animal at the same time. The level of anaesthesia, heart rate, blood pressure and temperature can all change between the two recordings and alter blood velocity and WSS. Physiological variability can lead to real differences in cycle length (*e.g.,* Fig. 7, last column). Such variation makes an exact match of WSS assessed by the two methods unlikely. Nevertheless, good agreement between CFUIV and CEUIV was observed (Figs. 6 and 7). We consider the absolute and relative differences between the measurements to be low. Accurate wall tracking is essential given that an offset of 200 *µ*m in wall location can lead to errors of up to 80% in WSS estimation (Leow and Tang 2018). The wall tracking algorithm used in this study has a mean absolute deviation <100 *µ*m (Riemer et al. 2020a). The accuracy of any assessment of WSS also depends on the spatial resolution of velocity measurement (Katritsis et al. 2007).

Assuming a Poiseuille flow profile, WSS is proportional to the mean velocity. For the comparison of CEUIV and CFUIV, flow and WSS were measured in a straight segment of the abdominal aorta where characteristics appear Poiseuille like. However, even in long straight segments of the aorta, the velocity profile might be skewed (Mynard et al. 2013). Measurement of WSS is clinically more useful in regions where flow is spatiotemporally varying and where analytic approximation fails. The low echogenicity and separability of slow flow might have a bigger impact in such situations.

### Limitations

Contrast-free ultrasound imaging velocimetry relies on the effective removal of clutter, and SVD clutter filtering, used in the present study, requires a sequence of images. It may not work in rapidly moving structures such as the heart. Buffering of images (Desailly et al. 2017), harmonic imaging (Paeng et al. 2010) or filtering methods based on machine learning may improve clutter removal and enable real-time CFUIV.

Like VFI, CFUIV can suffer from poor penetration depth. The depth of the rabbit abdominal aorta, imaged in this study, is equivalent to those of more superficial arteries such as the carotid and femoral, in people. In deeper vessels of the abdomen, in the brain or for cardiac imaging, contrast enhancement may be necessary to ensure a sufficiently high SNR.

Microbubble non-linear characteristics were neglected, and no contrast-specific or coded acquisition schemes were used, which can benefit CE and CF imaging, respectively. We also did not test different frequencies, which could be a useful parameter for optimising SNR and hence WSS accuracy. Finally, for practical reasons the MI was relatively low; a higher MI could further benefit CFUIV.

## Conclusions

In this study, we determined that CFUIV is capable of measuring spatially and temporally varying velocity and, hence, of assessing WSS, in large straight vessels *in vivo*. CFUIV is more easily applied than CEUIV, and provides similar accuracy if a sufficient SNR can be achieved.

## References

[bib0001] Baranger J, Arnal B, Perren F, Baud O, Tanter M, Demene C. (2018). Adaptive spatiotemporal SVD clutter filtering for Ultrafast Doppler Imaging using similarity of spatial singular vectors. IEEE Trans Med Imaging.

[bib0002] Badeau R, Richard G, David B. (2004). Sliding window adaptive SVD algorithms. IEEE Trans Signal Process.

[bib0003] Brands PJ, Hoeks AP, Hofstra L, Reneman RS. (1995). A noninvasive method to estimate wall shear rate using ultrasound. Ultrasound Med Biol.

[bib0004] Brown J, Christensen-Jeffries K, Harput S, Zhang G, Zhu J, Dunsby C, Tang MX, Eckersley RJ. (2019). Investigation of microbubble detection methods for super-resolution imaging of microvasculature. IEEE Trans Ultrason Ferroelectr Freq Control.

[bib0005] Cecchi E, Giglioli C, Valente S, Lazzeri C, Gensini GF, Abbate R, Mannini L. (2011). Role of hemodynamic shear stress in cardiovascular disease. Atherosclerosis.

[bib0006] Cloutier G, Qin Z, Durand LG, Teh BG. (1996). Power Doppler ultrasound evaluation of the shear rate and shear stress dependences of red blood cell aggregation. IEEE Trans Biomed Eng.

[bib0007] Collins RT, Laughlin ME, Lang SM, Bolin EH, Daily JA, Jensen HA, Jensen MO. (2019). Real-time transthoracic vector flow imaging of the heart in pediatric patients. Prog Pediatr Cardiol.

[bib0008] Correia M, Provost J, Tanter M, Pernot M. (2016). 4D ultrafast ultrasound flow imaging: In vivo quantification of arterial volumetric flow rate in a single heartbeat. Phys Med Biol.

[bib0009] Demené C, Deffieux T, Pernot M, Osmanski BF, Biran V, Gennisson JL, Sieu LA, Bergel A, Franqui S, Correas JM, Cohen I, Baud O, Tanter M. (2015). Spatiotemporal clutter filtering of ultrafast ultrasound data highly increases Doppler and fUltrasound sensitivity. IEEE Trans Med Imaging.

[bib0010] Desailly Y, Tissier AM, Correas JM, Wintzenrieth F, Tanter M, Couture O. (2017). Contrast enhanced ultrasound by real-time spatiotemporal filtering of ultrafast images. Phys Medic Biol.

[bib0011] Dhawan SS, Avati Nanjundappa RP, Branch JR, Taylor WR, Quyyumi AA, Jo H, Mcdaniel MC, Suo J, Coulter WH, Giddens D, Samady H (2010). Shear stress and plaque development. Expert Rev Cardiovasc Ther.

[bib0012] Du Y, Goddi A, Bortolotto C, Shen Y, Dell'Era A, Calliada F, Zhu L. (2020). Wall shear stress measurements based on ultrasound vector flow imaging. J Ultrasound Med.

[bib0013] Fadnes S, Nyrnes SA, Torp H, Lovstakken L. (2014). Shunt flow evaluation in congenital heart disease based on two-dimensional speckle tracking. Ultrasound Med Biol.

[bib0014] Feng J, Khir AW. (2010). Determination of wave speed and wave separation in the arteries using diameter and velocity. J Biomech.

[bib0015] Gates PE, Gurung A, Mazzaro L, Aizawa K, Elyas S, Strain WD, Shore AC, Shandas R. (2018). Measurement of wall shear stress exerted by flowing blood in the human carotid artery: Ultrasound Doppler velocimetry and echo particle image velocimetry. Ultrasound Med Biol.

[bib0016] Goddi A, Bortolotto C, Fiorina I, Raciti MV, Fanizza M, Turpini E, Boffelli G, Calliada F. (2017). High-frame rate vector flow imaging of the carotid bifurcation. Insights Imaging.

[bib0017] Hansen KL, Nielsen MB, Jensen JA. (2017). Vector velocity estimation of blood flow: A new application in medical ultrasound. Ultrasound.

[bib0018] He X, Ku DN, Moore JE. (1993). Simple calculation of the velocity profiles for pulsatile flow in a blood vessel using Mathematica. Ann Biomed Eng.

[bib0019] Holbek S, Christiansen TL, Stuart MB, Beers C, Thomsen EV, Jensen JA. (2016). 3-D vector flow estimation with row-column-addressed arrays. IEEE Trans Ultrason Ferroelectr Freq Control.

[bib0020] Hoskins PR. (2011). Estimation of blood velocity, volumetric flow and wall shear rate using Doppler ultrasound. Ultrasound.

[bib0021] Jensen JA. (1992). Calculation of pressure fields from arbitrarily shaped, apodized, and excited ultrasound transducers. IEEE Trans Ultrason Ferroelectr Freq Control.

[bib0022] Jensen JA. (1996). FIELD: A Program for Simulating Ultrasound Systems. Med Biol Eng Comput.

[bib0023] Jensen JA, Nikolov S, Yu AC, Garcia D. (2016). Ultrasound vector flow imaging: Part I. Sequential systems. IEEE Trans Ultrason Ferroelectr Freq Control.

[bib0024] Jensen JA, Nikolov S, Yu AC, Garcia D. (2016). Ultrasound Vector Flow Imaging - Part II: Parallel Systems. IEEE Trans Ultrason Ferroelectr Freq Control.

[bib0025] Katritsis D, Kaiktsis L, Chaniotis A, Pantos J, Efstathopoulos E P, Marmarelis V. (2007). Wall shear stress: Theoretical considerations and methods of measurement. Prog Cardiovasc Dis.

[bib0026] Kim HB, Hertzberg JR, Shandas R. (2004). Development and validation of echo PIV. Exp Fluids.

[bib0027] Lankton S. (2009).

[bib0028] Leow CH, Tang MX. (2018). Spatio-temporal flow and wall shear stress mapping based on incoherent ensemble-Correlation of ultrafast contrast enhanced ultrasound images. Ultrasound Med Biol.

[bib0029] Lupotti FA, Mastik F, Carlier SG, De Korte CL, Van Der Giessen WJ, Serruys PW, Van Der Steen AF. (2003). Quantitative IVUS blood flow: Validation in vitro, in animals and in patients. Ultrasound Med Biol.

[bib0030] Markou CP, Ku DN. (1991). Accuracy of velocity and shear rate measurements using pulsed doppler ultrasound: A comparison of signal analysis techniques. Ultrasound Med Biol.

[bib0031] Martin KH, Dayton PA. (2014). Current status and prospects for microbubbles in ultrasound theranostics. Wiley Interdiscip Rev Nanomed Nanobiotechnol.

[bib0032] Mohamied Y, Rowland E M, Bailey E L, Sherwin S J, Schwartz M A, Weinberg P D (2014). Change of direction in the biomechanics of atherosclerosis. Ann Biomed Eng.

[bib0033] Mynard JP, Wasserman BA, Steinman DA. (2013). Errors in the estimation of wall shear stress by maximum Doppler velocity. Atherosclerosis.

[bib0034] Nam KH, Yeom E, Ha H, Lee SJ. (2012). Velocity field measurements of valvular blood flow in a human superficial vein using high-frequency ultrasound speckle image velocimetry. Int J Cardiovasc Imaging.

[bib0035] Nguyen LC, Yu FT, Cloutier G. (2008). Cyclic changes in blood echogenicity under pulsatile flow are frequency dependent. Ultrasound Med Biol.

[bib0036] Paeng DG, Nam KH, Shung KK. (2010). Cyclic and radial variation of the echogenicity of blood in human carotid arteries observed by harmonic imaging. Ultrasound Med Biol.

[bib0037] Poelma C, Van Der Mijle RME, Mari JM, Tang MX, Weinberg PD, Westerweel J. (2012). Ultrasound imaging velocimetry: Toward reliable wall shear stress measurements. Eur J Mech B/Fluids.

[bib0038] Riemer K, Rowland EM, Leow CH, Tang MX, Weinberg PD. (2020). Determining haemodynamic wall shear stress in the rabbit aorta in vivo using contrast-enhanced ultrasound image velocimetry. Ann Biomed Eng.

[bib0039] Riemer K, Toulemonde M, Rowland EM, Leow CH, Tang MX, Weinberg PD. (2020). 4D blood flow and wall shear stress measured using volumetric ultrasound image velocimetry. Proc IEEE Int Ultrason Symp.

[bib0040] Rodríguez-Palomares JF, Dux-Santoy L, Guala A, Kale R, Maldonado G, Teixidó-Tura G, Galian L, Huguet M, Valente F, Gutierrez L, GonzalezAlujas T, Johnson KM, Wieben O, García-Dorado D, Evangelista A. (2018). Aortic flow patterns and wall shear stress maps by 4D-flow cardiovascular magnetic resonance in the assessment of aortic dilatation in bicuspid aortic valve disease. J Cardiovasc Magn Reson.

[bib0041] Rowland E, Riemer K, Lichtenstein K, Tang M, Weinberg P. (2020). P134 A new method for non-invasive measurement of arterial wave intensity, speed and reflection. Artery Res.

[bib0042] Sennoga CA, Yeh JSM, Alter J, Stride E, Nihoyannopoulos P, Seddon JM, Haskard DO, Hajnal JV, Tang MX, Eckersley RJ. (2012). Evaluation of methods for sizing and counting of ultrasound contrast agents. Ultrasound Med Biol.

[bib0043] Sheeran PS, Luois S, Dayton PA, Matsunaga TO. (2011). Formulation and acoustic studies of a new phase-shift agent for diagnostic and therapeutic ultrasound. Langmuir.

[bib0044] Stone PH, Saito S, Takahashi S, Makita Y, Nakamura S, Kawasaki T, Takahashi A, Katsuki T, Nakamura S, Namiki A, Hirohata A, Matsumura T, Yamazaki S, Yokoi H, Tanaka S, Otsuji S, Yoshimachi F, Honye J, Harwood D, Reitman M, Coskun AU, Papafaklis MI, Feldman CL. (2012). Prediction of progression of coronary artery disease and clinical outcomes using vascular profiling of endothelial shear stress and arterial plaque characteristics: The PREDICTION study. Circulation.

[bib0045] Trahey GE, Allison JW, von Ramm OT. (1987). Angle independent ultrasonic detection of blood flow. IEEE Trans Biomed Eng.

[bib0046] Voorneveld J, Kruizinga P, Vos HJ, Gijsen FJH, Jebbink EG, van der Steen AFW, de Jong N, Bosch JG. (2016). Native blood speckle vs ultrasound contrast agent for particle image velocimetry with ultrafast ultrasound - In vitro experiments. Proc IEEE Int Ultrason Symp IUS.

[bib0047] Wigen MS, Fadnes S, Rodriguez-Molares A, Bjåstad T, Eriksen M, Sten-sæth KH, Støylen A (2018). Lovstakken L. 4-D intracardiac ultrasound vector flow imaging-Feasibility and comparison to phase-contrast MRI. IEEE Trans Med Imaging.

[bib0048] Wikstrand J. (2007). Methodological considerations of ultrasound measurement of carotid artery intima-media thickness and lumen diameter. Clin Physiol Funct Imaging.

[bib0049] Yiu BY, Lai SS, Yu AC. (2014). Vector projectile imaging: Time-resolved dynamic visualization of complex flow patterns. Ultrasound Med Biol.

[bib0050] Yu FT, Franceschini E, Chayer B, Armstrong JK, Meiselman HJ, Cloutier G. (2009). Ultrasonic parametric imaging of erythrocyte aggregation using the structure factor size estimator. Biorheology.

[bib0051] Zhang F, Lanning C, Mazzaro L, Barker AJ, Gates PE, Strain WD, Fulford J, Gosling OE, Shore AC, Bellenger NG, Rech B, Chen JJ, Chen JJ, Shandas R. (2011). In vitro and preliminary in vivo validation of echo particle image velocimetry in carotid vascular imaging. Ultrasound Med Biol.

[bib0052] Zhou G, Zhu Y, Yin Y, Su M, Li M. (2017). Association of wall shear stress with intracranial aneurysm rupture: Systematic review and meta-analysis. Sci Rep.

